# Cannulated Screw Fixation For Femoral Neck Fractures : A 5-year Experience In A Single Institution

**DOI:** 10.5704/MOJ.1407.010

**Published:** 2014-07

**Authors:** CCH Khoo, Amber Haseeb,, Vivek Ajit Singh

**Affiliations:** Department of Orthopaedics, University Malaya Medical Centre, Kuala Lumpur, Malaysia; Department of Orthopaedics, University Malaya Medical Centre, Kuala Lumpur, Malaysia; Department of Orthopaedics, University Malaya Medical Centre, Kuala Lumpur, Malaysia

## Abstract

Cannulated screw fixation is a widely accepted surgical method for management of fractures of the neck of femur especially in patients with poor premorbid conditions, minimally displaced fractures and those from a younger age group. A five year retrospective study was carried out in 53 consecutive patients between 2006 to 2010 to determine the pattern of injuries, management, outcomes and the associated predictive factors.All the patients underwent cannulated screw fixation, with 37 (69.8%) having had surgery within 24 hours and the remaining 16 (30.2%) 24 hours after the initial injury. All patients were followed up to union of fractures and complications thereafter if any. Good outcome was observed in 43 (81.1%) patients leaving only 10 (18.9%) patients with a poor outcome, of whom nine developed avascular necrosis (90%) and one non-union (10%). We found no significant relationship between the incidence of avascular necrosis and age of patient, fracture displacement, numbers of cannulated screws used, fracture reduction acceptability and anatomical location of the fracture. The time interval from injury to surgery and the presence of posterior comminution did seem to influence the rate of avascular necrosis but due to the small number of patients, was not statistically significant.We conclude that cannulated screw fixation is a viable option of treatment for fractures of the neck of femur.

**Key Words:**

femoral neck fractures, screw fixation

## Introduction

Fracture of the neck of femur is a challenging injury to manage. It is one of the more common injuries presenting to the emergency room and is likely to remain so in the near future. It is often a fracture of fragility due to osteoporosis in the elderly, though in the younger age group, it usually results from high-energy trauma sustained in a road crash. Internal fixation remains the treatment of choice for these fractures in all age groups, more so in displaced fractures in the younger patients, where preservation of the femoral head is the priority. However, the optimal timing for surgical fixation of these fractures is still open to debate. It is advocated that fracture reduction and fixation should be performed as a surgical emergency in an attempt to restore the precarious blood supply to the femoral head and prevent complications such as non-union and avascular necrosis, the incidence of these complications being 10-20% and 10-30% respectively ^1,2,3,4^. Non-union and avascular necrosis predisposes to future degenerative arthritis of the hip joint involved. Revision surgery or conversion surgery to hip replacement is reported to be between 20-36% ^5,6^.

We conducted a study to evaluate the outcome of cannulated screw fixation for femoral neck fracture in our institution. Special attention was given to the time lapse from injury to surgery in relation to union and occurrence of complications such as avascular necrosis and non-union. The aims were to determine if there was any difference between early surgical fixation (less than 6 hours) and delayed fixation (6 hours and more), the prognostic factors for occurrence of avascular necrosis and finally the conversion rate to hip replacement in our patients who eventually developed complications.

## Materials and Method

This is a retrospective review of all cases of femoral neck fractures that underwent cannulated screw fixation at our centre between the years 2006 till 2010. Review of all case records was carried out with the aid of a questionnaire and the radiographs by three independent observers, including a senior orthopaedic consultant.

The fractures in all our patients were analysed for displacement and stability, using the Garden Classification and Pauwel Classification respectively. Garden’s Alignment Index was used to assess fracture reduction postoperatively, with 155° - 180° in both antero-posterior and lateral view as the criterion - for adequate reduction.

**Operative Procedures** Emergency closed reduction and percutaneous cannulated screw fixation of the femoral neck fractures were performed in all our patients under either general - or regional spinal or epidural anaesthesia. During the procedure, the patient was positioned supine on a traction table with the foot secured to the footplate. The fracture was then visualised with an image intensifier. Undisplaced fractures were fixed in-situ, while displaced fractures were reduced by closed manipulation by first externally rotating the hip joint followed by abduction, then applying longitudinal traction to the limb and the fracture subsequently reduced by internal rotation and adduction of the hip joint. Three standard cannulated (7.3mm cancellous) screws were inserted for all our patients, following the “3 Point Principle” except in cases where the femoral neck was small in diameter and could only accommodate two screws. Hip joint capsulotomy was not performed in any of the cases.

**Follow up** Post operatively, the patients were advised to ambulate strictly with non-weight bearing crutches until there was radiological evidence of union. Post-operative hip radiographs were taken and these were subsequently analysed for acceptability of fracture reduction and fixation using the Garden’s Alignment Index. These patients were then followed up till there was evidence of radiological union. Documented incidence of non-union and avascular necrosis were noted and analysed individually in relation to the following risk factors: Age of patient, elapsed time between injury and surgery, presence of posterior comminution, fracture displacement at presentation. number of cannulated screws used, fracture reduction acceptability, anatomical location of fractures.

Age of patient was taken as the age on day of admission to the hospital. Elapsed time was calculated from time of injury to starting time of surgery. Posterior comminution was detected either during surgery under image intensifier or post operatively in plain radiographs as it is sometimes not easy to detect on initial radiograph. Fracture displacement was classified as either displaced (Garden Classification of neck of femur fracture Type III and IV) or undisplaced (Garden Classification of neck of femur fracture Type I and II). Fracture reduction acceptability was assessed using Garden Alignment Index by measuring the angle of the compression trabeculae on AP and lateral view relative to the longitudinal axis of the femoral shaft and considered acceptable if it fell within the 155° - 180° range. Anatomical location of fracture was divided into subcapital, transcervical or base of neck.

Avascular necrosis (AVN) of the femoral head was diagnosed based on radiographic evidence and clinical features, using the Ficat & Arlet Classification (Table I).

Good outcome was defined radiologically as fracture union with no evidence of non-union or avascular necrosis. Statistical Analysis was performed using Pearson Chi Square Test and Fisher’s Exact Test. A p value of less than 0.05 was considered to be statistically significant.

**Fig. 1: Incidence of Avascular Necrosis Per Age Range.**
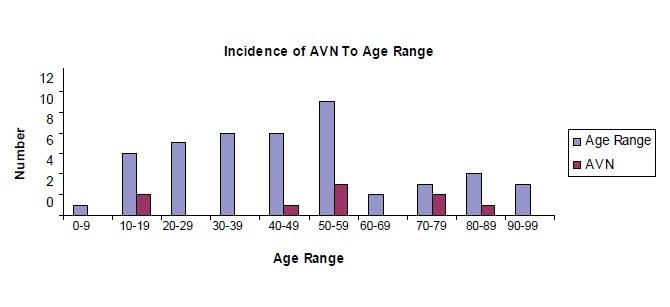


**RESULTS**There were 53 cases identified during the study period. 39 patients were males (73.6%) and 14 were females (26.4%). Majority of the patients were from 30 to 59 years of age with a mean age of 42.1 years (age range 6 to 91 years), as shown in figure 1. Majority (36 patients) of our patients were victims of road crash and the remainder (17 patients) had falls.

Radiographs taken on admission showed 16 cases of basal neck fractures, 10 subcapital -and 27 transcervical. Thirtysix patients (67.9%) had displaced fractures (Garden III or IV) and 17 (32.1%) had undisplaced fractures (Garden I or II). Fractures angle of inclination was Pauwels 1 (<30°) in 6 patients, Pauwels 2 (30° - 50°) in 30 patients and Pauwels 3 (>50°) in 17 patients. All of them underwent closed reduction and percutaneous cannulated screw fixation. Twenty-five patients had surgery within the first 12 hours of the injury while the rest (28 patients) underwent surgery more than 12 hours after injury, of whom 16 had their surgery more than 24 hours after the initial injury. Majority (35 patients) had the standard three screws fixation while 18 patients had only two screws - due to the size of the femoral neck.

**Fig. 2: Interval to Surgery and Incidence of AVN.**
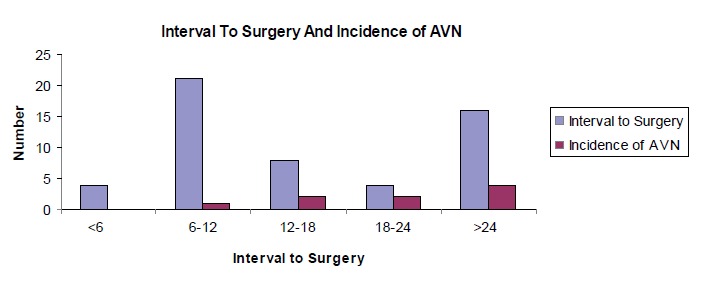


Fracture union occurred in 52 patients with a mean time to union of 3.74 months (range 1.5 months to 8 months), Nine patients developed avascular necrosis of the femoral head. Incidence of avascular necrosis according to age range is illustrated in Figure 2. There was one case of non-union.

Forty-three patients had good clinical outcome at the end of the review with no documented evidence of avascular necrosis or non-union. Four out of the nine patients with avascular necrosis eventually were planned for conversion surgery to total hip replacement for symptomatic osteoarthritis. The conversion rate to total hip replacement in our review was 7.55% (4 of 53 patients). However only three patients eventually underwent total hip replacement, one patient opted for conservative treatment due to advanced age. The other five cases of avascular necrosis had mild to moderate osteoarthritis not needing a hip replacement at the time of review. Table II shows the characteristics of the nine patients who had avascular necrosis.

In our study the patients who had conversion surgery to total hip replacement were generally 60 years or above in age and had pre-existing co-morbidities; . 75% of patients were in this category.

The patient with non-union was a 53 years old lady who had sustained a transcervical femoral fracture (Gardens 2 and Pauwel 2) after a road crash She was treated with cannulated screw fixation after more than 24 hours after the injury due to unavoidable circumstances. After six months of follow up, vascularised bone grafting was done and the fracture finally united after 26 months from the time of injury.

## RESULTS

The overall incidence of avascular necrosis in our study was 16.98% (9 of 53). Incidence of avascular necrosis as per age range is shown in Table II. We found no significant association between age of patient and avascular necrosis (p = 0.462).

The relationship between interval to surgery and incidence of avascular necrosis in our patient is shown in Figure 2. We found that the incidence of AVN increased with increasing time lapse. There was no incidence of AVN in cases operated less than 6 hours but it gradually increased as the time interval increased. The incidence of AVN for surgery done less than 24 hours was 13.51% as compared to 25% for surgery done later than 24 hours. The incidence of AVN compared between cases where the surgery was done before 6 hours and after 6 hours, was statistically insignificant (p = 0.5).

**Fig. 3: Incidence of AVN in Relation to Location of Fracture.**
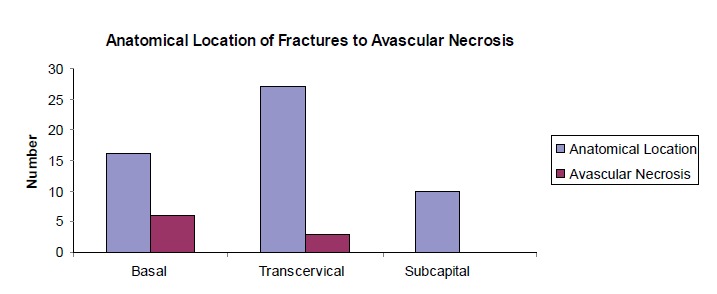


All the patients in our study had sustained intracapsular fractures. Upon analysing the relationship of location of fractures whether base of neck, transcervical or subcapital , we found no statistical significance between locations of fractures and avascular necrosis (p = 0.096). The incidence of avascular necrosis in fractures at base of neck was 37.5% (6 of 16); transcervical fracture 11.11% (3 of 27), while all subcapital fractures united without any incidence of avascular necrosis (Figure 3).

**Fig. 4: Incidence of AVN to Integrity of Posterior Cortex.**
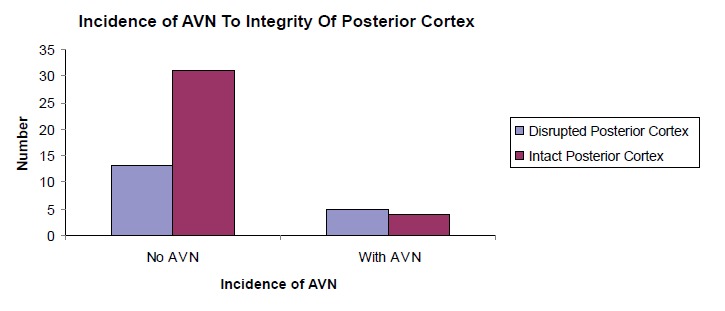


Posterior comminution was present in 18 of our patients (33.96%) while 35 cases had an intact posterior cortex (66.04%). 27.78% of patients with posterior comminution developed avascular necrosis while only 11.43% of patients without posterior comminution developed avascular necrosis (Figure 4). However, the relationship between disrupted posterior cortex and incidence of avascular necrosis is not statistically significant (p = 0.108).

The incidence of AVN in relation to numbers of cannulated screws used during the surgical fixation was 5.6% (1 of 18 patients) for two screws compared to 22.9% (8 of 35 patients) for three screws. However, this relationship is not statistically significant (p = 0.112).

**Fig. 5: Adequacy of Fracture Reduction to Risk of Avascular Necrosis.**
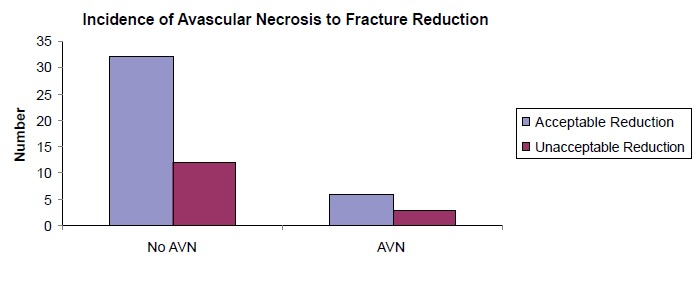


Adequacy of fracture reduction with subsequently stable fixation was achieved in 71.7% (38 of 53 patients) while inadequate reduction occurred in 28.3% (15 of 53 patients). Figure 5 illustrates the incidence of avascular necrosis in relation to adequacy of fracture reduction. There were six cases of AVN in the adequately reduced group (16 %) as opposed to three cases of AVN in the inadequately reduced group (20 %). We found no significant relationship between adequacies of fracture reduction and risk of avascular necrosis (p = 0.774).

Displaced fractures (Garden III and IV ) occurred in 36 patients (67.92%) while 17 patients (32.08%) had undisplaced fractures (Garden I and II ). We found no statistically significant relationship between displacements of fractures and incidence of avascular necrosis in our study (p = 0.487). Avascular necrosis developed in 19.44% of displaced fractures compared to 11.76% in undisplaced fractures.

## Discussion

Fractures of femoral neck remain a challenge in the clinical practice of orthopaedic surgeons. It is still a subject of debates over the years with regards its management. Generally, the accepted mode of treatment is internal fixation either by open or closed reduction in younger patients and patients without degenerative changes in the hip joint. Total hip replacement is generally a preferred option for patients with pre-existing degenerative changes in the hip. The primary aim of internal fixation in these fractures is to achieve anatomical reduction in order to restore or preserve the precarious blood supply to the head of femur, more so in the younger age group and prevention of future complications such as avascular necrosis and non-union. Stable anatomical reduction can be achieved by means of three standard cannulated screws (7.3mm cancellous) inserted according to the “3 Point Principle”.

The overall incidence of avascular necrosis in our series was 16.98%, which is comparable with the majority of previous published data (ranging from 10% - 30%) ^1, 2, 3, 4^. Avascular necrosis of the head of femur leads to segmental collapse of the head which predispose to secondary hip joint degenerative changes, necessitating subsequent revision or joint replacement surgery.

Previous studies have shown contradicting findings with regards the relationship between age of patient to subsequent incidence of avascular necrosis. The traditional belief is that there is decreasing risk of avascular necrosis with increasing age, as shown by Graham ^8^, Barnes ^9^ and Luizou ^10^ . However, Shih & Wang ^11^, concluded that there was no significant association between age of patient and the incidence of avascular necrosis developing later on in life. Our findings seem to correlate with the conclusion of Shih & Wang as there was no significant association between age and incidence of avascular necrosis.

Urgent reduction and stable fixation of femoral neck fracture less than six hours after injury has been shown to reduce the risk of avascular necrosis ^12^. Jain reported that time to reduction of the fracture was found to be the only significant contributing factor, though others have shown no difference between early or later surgery ^13^. In our study, we found no cases of AVN when the fracture was reduced and fixed within six hours but the incidence increased with increasing time interval. However this is not statistically significant due to the small number of cases. Therefore, there is still a case for urgency in getting these patients to surgery and stabilising these fractures.

Majority of the blood supply to the femoral head comes from the medial and lateral femoral circumflex arteries with minimal contribution from the obturator vessels ^14, 15^. The medial and lateral femoral circumflex arteries arise from superficial femoral artery and curl around the trochanteric region before branching proximally - to supply the head. Our findings found no significant relationship between locations of fractures to incidence of avascular necrosis. However, we noticed that base of neck fractures had a 37.5% chance of developing avascular necrosis as compared to 11.11% incidence of avascular necrosis in transcervical fractures. More importantly, all 10 cases of subcapital fractures in our series – did not develop subsequent avascular necrosis. The transcervical region and the basal region of the neck is the watershed area between the blood supply from the femoral head and the shaft of the femur, and therefore having a relatively poorer blood supply. Once the epiphysis is closed, there is no more intra-osseous anastomosis between branches of epiphyseal artery and metaphyseal artery, leaving a potential watershed zone within the subcapital region. Furthermore, in both transcervical and basal neck fractures, the segment proximal to fracture line up to the subcapital region evolves to contribute to a watershed zone as it is known that this segment is intracapsular and therefore has no periosteum.

The posterior cortex of the femoral neck plays an important role in the stability of femoral neck fractures. Its integrity is proposed as an important factor for successful surgical management of subjects with femoral neck fracture of all ages. Posterior comminution is a risk factor in failed internal fixation even when adequate reduction is achieved during initial surgery. Additionally, the course of the medial femoral circumflex artery around the posterior cortex of the femoral neck exposes it to risk of injury in femoral neck fracture with severe posterior cortical comminution. As mentioned earlier, the branches of this important vessel supply the superolateral part of the head predominantly. With disruption of the blood supply to this weight bearing area of the head due to posterior cortical comminution, there is documented increased risk of avascular necrosis Huang et al ^16^ showed that disrupted posterior cortex increased the risk of avascular necrosis in their patients. They went further to show that patients who had fracture of femoral neck with posterior cortical disruption were more likely to end up with revision surgery, usually joint replacement, either bipolar or total hip. This was also observed by other authors such as Frangakis ^17^, Scheck ^18, 19^ in his papers in 1959 and 1980, and more recently, by Alho et al ^20^ in 1993. In our study, we noticed that the incidence of avascular necrosis in the presence of posterior comminution is almost 2.5 times more compared to those without posterior comminution.

One of the most important issues during closed reduction and percutaneous cannulated screw fixation is the adequacy of fracture reduction and subsequent stable fixation. Adequate fracture reduction is assessed using the Garden Alignment Index. This index refers to the angle of the compression trabeculae on AP view relative to the longitudinal axis of the femoral shaft and the angle of the compression trabeculae on the lateral view relative to the femoral shaft. In a normal radiograph of the hip joint, this angle should be 160° on the AP view, while on the lateral view it is 180°. Acceptable reduction is defined as a reduction angle lying within 155°-180° range on both views. It is universally accepted that risk of avascular necrosis increases substantially if the alignment index falls out of this acceptable range, especially if there is a valgus reduction of more than 20 degrees. The incidence of avascular necrosis in patients with adequate fracture reduction in our study was 15.79%, comparable to 20% for those with inadequate fracture reduction. This is however not statistically significant.

We have described the precarious course of the blood supply to the head of femur and the intimate relationship along the neck to the head from the trochanteric region of the femur. A severely displaced fracture may rupture or stretch these important vessels, thus putting the head at risk of ischemia.

Strong relationship is reported in the literature between the risks of avascular necrosis in displaced intracapsular fractures compared to undisplaced femoral neck fractures ^9, 11, 21^. Asnis & Wanek-Sgaglione ^22^ reported an incidence of avascular necrosis of almost 20% in undisplaced fractures (Garden II) in their study. We found no significant difference between displaced and undisplaced fracture with regard to future risk of avascular necrosis in our study. We postulate that the initial force sustained at the proximal femur during the causal injury is directly related to the fate of the head of femur in terms of future avascular necrosis. The vascularity of the head could have been disrupted from the initial trauma, therefore the adequacy of reduction did not influence the rate of avascular necrosis. Tooke & Favero KJ ^23^ and Protzman & Burkhalter ^24^ also echoed similar view in their reports.

The conversion rate to total hip arthroplasty in patients who developed avascular necrosis in our series was 7.55%. We noticed that the typical patient who underwent conversion surgery was usually above 60 years of age and had multiple pre-existing co-morbidities. We feel that even though cannulated screw fixation remained a good option of treatment for neck of femur fracture, as shown by the good outcome in our series, total hip replacement should be considered in this particular group of patients.

As this is a retrospective study documenting a five year experience in the treatment of intracapsular neck of femur fracture by closed reduction cannulated screw fixation, the follow up period for the patients was not standardised. Therefore the incidence of avascular necrosis could have been underestimated. Secondly, the number subjects are relatively small, compared to other studies and this could have affected the conclusions. Lastly, some cases of early avascular necrosis of the head of femur might not have been picked up as Magnetic Resonance Imaging was not used for these cases.

**Table I: Classification of Ficat & Arlet7:**
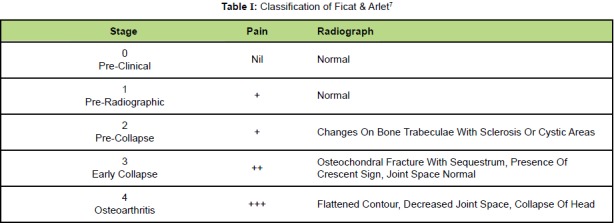


**Table II: Characteristics of Patients Who Developed Avascular Necrosis:**
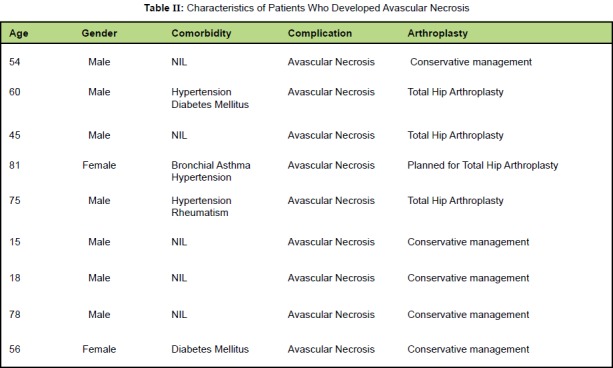


## Conclusion

In our five years’ experience in treating neck of femur fracture with closed reduction and cannulated screw fixation, we found no significant relationship between the incidence of avascular necrosis in relation to age of patient, fracture displacement, number of cannulated screw used, fracture reduction acceptability and anatomical locations of fracture. The time interval from injury to surgery and the presence of posterior comminution did influence the rate of avascular necrosis but due to the small numbers in the study, it is not statistically significant. In conclusion, cannulated screw fixation remains a viable option of treatment for neck of femur fracture.
